# Evaluating the interstitial lung disease multidisciplinary meeting: a survey of expert centres

**DOI:** 10.1186/s12890-016-0179-3

**Published:** 2016-02-01

**Authors:** Helen E. Jo, Tamera J. Corte, Yuben Moodley, Kovi Levin, Glen Westall, Peter Hopkins, Daniel Chambers, Ian Glaspole

**Affiliations:** 1 Royal Prince Alfred Hospital, Sydney, Australia; 2 University of Sydney, Sydney, Australia; 3 University of Western Australia, Perth, Australia; 4 Fiona Stanley Hospital, Perth, Australia; 5 Respiratory Health Institute, Perth, Australia; 6 The Alfred Hospital, Melbourne, Australia; 7 Monash University, Melbourne, Australia; 8 Prince Charles Hospital, Brisbane, Australia; 9 University of Queensland, Brisbane, Australia

**Keywords:** Idiopathic pulmonary fibrosis, Interstitial lung disease, Multidisciplinary meeting, Diagnosis

## Abstract

**Background:**

Multidisciplinary meetings (MDM) are the current “gold standard” in interstitial lung disease (ILD) diagnosis and comprise inter-disciplinary discussion of multiple forms of information to provide diagnostic and management outputs. Although bias could be potentially inserted at any step in the discussion process, to date there has been no consensus regarding the appropriate constitution and governance of MDM. We sought to determine the features of ILD MDMs based within ILD centres of excellence around the world.

**Methods:**

An internet based questionnaire was sent to twelve expert centres in Europe, North America, and Australia seeking information regarding the structure and governance of their MDM. Data was analysed for consistent themes and points of contrast.

**Results:**

Responses were received from 10 out of 12 centres. Similarities were demonstrated with regards to contributing attendees, meeting frequency and case numbers reviewed. Significant heterogeneity in attendee speciality group type, quantity and method of data presentation, approach to diagnosis formulation and documentation, and information provision was apparent.

**Conclusions:**

The constitution of ILD MDMs differs considerably between expert centres. Such differences may result in discordant outcomes, and emphasise the need for further evidence regarding the appropriate constitution and governance of ILD MDMs.

**Electronic supplementary material:**

The online version of this article (doi:10.1186/s12890-016-0179-3) contains supplementary material, which is available to authorized users.

## Background

Differences in clinical presentation of interstitial lung diseases (ILD) can be subtle, yet their natural history and response to therapy may display striking disparity [1, 2]. For example, idiopathic pulmonary fibrosis (IPF), a progressive and fatal subtype of idiopathic interstitial pneumonia (IIP), displays clinical, radiological and histological features that have considerable overlap with other subtypes. While IPF historically possesses a significantly worse prognosis [3], anti-fibrotic therapies are now available that delay its progression [4, 5], making its accurate identification critical. With the use of a multidisciplinary meeting (MDM), the differentiation of IPF from other diagnoses occurs with improved interobserver agreement along with improved recognition of rare diseases [6]. As a result of this, recent consensus statements regarding IPF and ILD have advocated that a multi-disciplinary approach be used for their diagnosis [2, 7, 8].

By definition, the MDM requires input from a variety of specialties, but the most appropriate form of MDM constitution and governance remains unclear. The constitution of ILD MDMs may have important diagnostic and therapeutic implications, Flaherty et al. demonstrating that academic physicians in a multidisciplinary setting display better diagnostic agreement and consider a broader range of diagnoses, compared to community physicians [9]. The ILD MDM has potential for bias depending on the expertise and number of its attendees, nature of clinical inputs and quality and quantity of data provided. Such biases may have profound effects should they lead to diagnostic error, given that resultant therapeutic decisions may, for example, lead to the use of agents for indications where efficacy is not established, or even for those where they may cause harm [10]. We sought to determine the constitution and governance of ILD MDMs in expert centres to provide information on current standard of care.

## Methods

Following approval by the Alfred Hospital (Melbourne, Australia) ethics committee (project number 65/15), an internet based questionnaire was sent to twelve expert centres in Australia, Europe, and North America, using SurveyMonkey™ (Palo Alto, USA). All respondents provided written informed consent for participation in the study. Purposive sampling was performed to ensure multiple continents were included among the responders. Within those regions, expert centres were defined as those to which the affiliated clinician had most frequently published in the field of IPF in 2014 as determined by their publication count within the US National Library of Medicine online database. We did not differentiate between ILD specific centers and larger hospitals, nor whether there were some patients within those institutions who were not presented at the MDM. Surveyed physicians were asked to describe their affiliated institution’s MDM using a questionnaire that included the opportunity for respondents to provide both prompted and open-ended answers (Additional file 1). Responses were grouped to establish points of consistency and contrast among expert centres. Topics included MDM organisation and structure, constitution, diagnostic methodology and information provided to referring physicians.

## Results

After an initial request and one reminder 5 days later, responses were received from ten out of twelve expert ILD centres (83 %). Centres were located in Australia, the USA, Canada, the United Kingdom and France.

### Organisation and structure

All centres had dedicated ILD MDMs with most centres meeting once every 1–2 weeks (90 %). All but 1 centre discussed six or more cases per meeting, with 4 centres discussing greater than 10 cases per meeting. The median duration of the meetings was 31 to 60 min although 3 centres had meetings greater than 90 min.

All MDMs were attended by thoracic clinicians, radiologists and pathologists, and these members contributed most to the MDM. In most centres (90 %), the MDM was also attended by junior staff and many had nursing staff present (80 %), with variable contribution from these groups. In a minority of MDMs, there was attendance by rheumatologists (30 %), thoracic surgeons (20 %) and transplant physicians (30 %). When present, rheumatology and palliative care physicians contributed always or frequently to the MDM discussion (Table 1).

**Table 1:** Attendance and contribution of MDM members

Specialty	Attendance	Always contributes	Frequently contributes
Thoracic clinician	100 %	100 %	
Radiologist	100 %	90 %	10 %
Histopathologist	100 %	100 %	
Trainees	90 %		67 %
Nursing staff	80 %		36 %
Rheumatologist	30 %	33 %	67 %
Transplant physician	30 %	33 %	33 %
Thoracic surgeon	20 %		33 %
Immunologist	10 %		
Palliative care	10 %	100 %	

### Governance

**Fig. 1:**
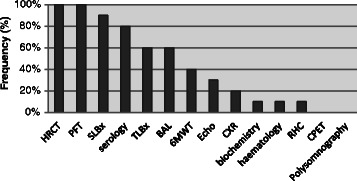
Minimum requisite investigations presented at ILD MDMs

Heterogeneity in the extent and format of information presented at the ILD MDMs was apparent. In 60 % of ILD MDMs, only select clinical and investigation findings that were thought to be relevant by the referring team were presented. The remaining 40 % of meetings presented all findings and 30 % of meetings used a uniform template as the basis for presentation. 60 % presented clinical data in an audiovisual presentation and the remaining 40 % used only an oral summary. All centres required a high resolution CT chest and pulmonary function tests and most required a surgical lung biopsy (if performed) (90 %) and rheumatologic serology (80 %). Six minute walk test and echocardiography was required in 40 and 30 % of MDMs respectively (Fig. 1).

Thoracic physicians adopted the dominant role in MDM. In 90 % of meetings, discussion was led by thoracic physicians, and 100 % of respondents reported that physicians were the accountable craft group for the diagnosis once it had been made. The referring physician often had a major role in the MDM. In 70 %, the referring physician was responsible for documenting the diagnosis, and in 60 % the referring physician had the greatest role in formulating the diagnosis. The governance of diagnostic dilemmas varied between institutions. While in 60 % of MDMs, a consensus approach to diagnosis was used, in 30 % the final decision was left to the clinician responsible for the case. In one centre, the final diagnosis was made by the chair of the MDM. Table 2 displays the commonest diagnostic dilemmas facing expert MDMs.

**Table 2:** Common diagnostic dilemmas

Dilemma type	Total responses	First listed dilemma
Diagnostic dilemmas		
IPF diagnosis, classification and differentiation from other ILDs	8	6
Recognition of chronic HP	6	5
Recognition of non-IPF ILD’s: CTD-ILD, CPFE, cystic lung disease, NSIP	9	4
Interpretation of poorly classifiable findings	4	0
Interpretation of poor quality diagnostic material	2	0
Management decisions		
Need for biopsy	2	0
Whether to recommend immune-suppression	3	0
Whether to recommend anti-fibrotic therapy	2	0

### Information generated

All MDMs delivered a diagnosis and differentials with 80 % also including a degree of diagnostic confidence. A minority of MDMs also included a prediction of disease behaviour (30 %). ILD MDMs generated management recommendations and treatment aims in 80 and 60 % of MDMs respectively. Responses are categorized in Table 3 according to their degree of heterogeneity.

**Table 3:** Differences in surveyed Multidisciplinary meetings (MDM)

Similarities	Minor differences	Major differences with potential to introduce bias
Organization and structure		
Exclusively ILD MDM 1–2weekly MDM >6 case load 31–60 min durations Attendance by thoracic physician, radiologist, histopathologist, junior staff, nursing staff.	3–4 weekly MDM >10 case load >90 min duration	Attendance by rheumatologist, immunologist, transplant physician, thoracic surgeon.
Governance		
Performance of HRCT, PFT, lung biopsy, rheumatological serology. MDM lead by thoracic physician	Use of audiovisual presentation vs oral presentation only	Presentation of relevant vs all clinical findings. Presentation using a standard template vs no template
Information provision		
Diagnosis and differentials Diagnostic confidence	Clinical behaviour classification Treatment aims	Final diagnosis made by consensus vs clinician responsible for case

## Discussion

Our survey has shown that significant heterogeneity exists in MDM constitution, governance, data input, diagnostic process and information provision. The impact of these differences is unclear, but potentially of crucial importance were it to lead to inaccuracy in disease recognition and resulting therapeutic choices. Also, as MDM diagnosis becomes the clinical benchmark, with access to medications restricted by MDM diagnosis, it is important that we recognise the limitations of the MDM and highlight methods to improve consistency and accuracy.

No randomised trial has ever been performed, or is ever likely to be, that demonstrates MDM based diagnosis results in improved patient survival. Its utility is instead based on the “linked evidence approach” [11] whereby benefit is derived through improved diagnostic accuracy and consequent alterations in therapeutic approach. It is clear that there is an increasing range of therapeutic choices for ILD, including anti-fibrotic therapy for IPF [4, 5], antigen avoidance for chronic hypersensitivity pneumonitis [12, 13], immune suppression for inflammatory and connective tissue disease related disease [14–20], lung transplantation [21] and palliative approaches [7]. Many of those therapies have robust evidence for their benefit when applied to specific diagnoses, such as the anti-fibrotic therapies nintedanib and pirfenidone [4, 5] which are now recommended therapies in the revised IPF consensus statement [22, 23]. Given the established evidence for MDM’s association with improved diagnostic accuracy [2, 6, 24], our finding that MDMs are a ubiquitous feature among the expert centres surveyed in our study is not a surprising finding.

Despite the clear utility and importance of ILD MDMs, the constitution and governance of these meetings has never been explored or addressed. Our survey shows that expert centres’ MDM demonstrate several points of consistency in their meetings, being constituted at a minimum by thoracic physicians, radiologists and pathologists - although contribution from non-thoracic clinicians varied - and considering a similar set of clinical and investigative data. However, within this range of broad similarity, significant heterogeneity was seen in MDM governance. This was apparent in the extent of information provided, the presentation style and the approach to resolution of diagnostic dilemmas, which varied between a consensus approach and deferral to the clinician responsible for the case. While the effects of such heterogeneity amongst ILD MDMs are not clear, it could be hypothesised that those with less information presented and less group input with regard to final diagnosis would behave little differently to an individual clinician. Further research that explores the effects of such heterogeneity on MDM diagnostic performance, considers MDM models that provide the most accuracy and concordance, validates the utility of MDM versus other models of diagnosis, and longitudinally explores outcomes, is of paramount importance if MDM is to adequately perform a role as a final arbiter of diagnosis.

Our survey also demonstrated that the majority of MDMs generate outputs in addition to diagnosis, including management recommendations and treatment aims. Interestingly, while multi-craft group meetings focused both on diagnosis and management are the norm in the treatment of other pulmonary conditions such as lung cancer, in only a minority of ILD MDMs did other craft groups attend that might contribute to forming those additional outputs. This is despite the fact that many ILD diagnoses have co-morbidities, and produce significant impairment and mortality, such that the meeting may provide an excellent opportunity for referral to relevant craft groups, such as transplant [21] and palliative care services [7]. The utility of non pulmonary craft group attendees was highlighted in our survey by rheumatology attendees, who contribute frequently or always to discussion in 100 % of meetings they attended.

Clearly, local regulatory and insurer factors will have significant impact on the frequency and nature of the use of MDM in the management of ILD, and any position statement would need to be tailored to reflect regional circumstances. For those regions where MDM is used, we propose a set of core criteria for structure and governance, given our survey’s findings with regards to heterogeneity and the implied impact this may have on diagnosis and subsequent therapeutic recommendation. These are likely to include the features listed in Table 4, which were common to the majority of centres surveyed. A number of other features, including the attendance and influence of other specialties, presentation format, and data outputs other than a diagnosis, were not uniform, and thus are difficult to ascribe as core features. However, in developing any expert statement, we suggest such features be considered and guidance given as to which additional features to core criteria are preferred, particularly those that might decrease potential bias and increase the utility of the ILD MDM.

**Table 4:** Core criteria for interstitial lung disease multidisciplinary meetings

1. An adequate case-load to enable a frequency of meetings commensurate with the development and maintenance of expertise in ILD diagnosis; 2. Attendance by at least one respiratory physician, radiologist and histopathologist; 3. Data presentation by the clinician directly responsible for the patient’s care; 4. Presentation of a set of routine investigations that include high quality HRCT images, PFT, rheumatological serology and, if available, histology; 6. A consensus approach to diagnosis formulation; 7. The provision of a diagnosis, degree of diagnostic confidence, and differential diagnoses.	

An important area our study did not explore is the prescription patterns for those patients undergoing diagnosis via heterogeneous MDMs. Our study focused solely on expert centres, but it is impractical to imagine that in most regions there are sufficient expert centres present to provide similarly constituted MDM. It is therefore inevitable that heterogeneity will exist in MDM with regards to expertise, based on whether they are located in community or academic centres. Academic MDMs when compared to community centres have previously been demonstrated to possess different diagnostic performance, with an increased likelihood of alternative diagnoses to IPF and therefore by corollary, a probable decreased frequency of prescription of anti-fibrotic therapy [9]. A further contributor to such differences in prescription rates may be via regional differences in approaches taken by regulatory authorities to anti-fibrotic therapy prescription and whether or not the MDM has been specifically mandated to control the prescription of anti-fibrotics. Each of these potentially has fundamental health economic implications, with regards the frequency of prescription of anti-fibrotic therapies.

A perceived limitation of our study is its concentration solely on expert centres. These centres however, were deliberately chosen in the belief they would most likely have a common approach to ILD diagnosis and management. Despite being a small and highly selected sample, the centres demonstrated significant heterogeneity. A wider survey of non-academic centres to determine whether even broader heterogeneity in MDM output occurs would be worthwhile. Also, we did not explore any specific patient outcomes but instead inferred using existing literature that heterogeneity in ILD MDMs would likely result in a significant difference in ILD MDM diagnostic performance [6, 9, 24–28]. This hypothesis requires confirmation in further studies that explore differences in diagnosis and treatment recommendations.

## Conclusion

We have demonstrated that ILD MDMs in expert centres differ considerably in their organization and structure, governance and information generated. While ILD MDMs are the “gold standard” for diagnosis, there are limited data on the impact that heterogeneity in MDMs has on diagnostic and management outputs. There are indications that meeting format and the expertise of attendees alters diagnostic agreement but further research to clarify the concordance between heterogeneous MDMs using current diagnostic guidelines is required. As ILD MDMs become increasingly frequent and produce additional outputs, there is a need for evidence based clinical guidelines regarding their constitution and governance to ensure the best clinical outcomes in these frequently devastating diseases.

## Additional file

Additional file 1:
**Survey Questions.** (DOCX 28 kb)

## Abbreviations

IPF: idiopathic pulmonary fibrosis
IIP: idiopathic interstitial pneumonia
ILD: interstitial lung disease
MDM: multidisciplinary meeting
UIP: usual interstitial pneumonia
HRCT: high resolution computed tomography
PFT: pulmonary function test
6MWT: 6 min walk test
TTE: transthoracic echocardiogram
RHC: right heart catheterisation
CPET: cardiopulmonary exercise testing
TLBx: transbronchial lung biopsy
BAL: bronchoalveolar lavage
