# Supplementary material for: Evaluating the interstitial lung disease multidisciplinary meeting: a survey of expert centres
Source: BMC Pulm Med. 2016 Feb 1;16:22. doi: 10.1186/s12890-016-0179-3 (PMC4736654; doi:10.1186/s12890-016-0179-3)
Supplement: Additional file 1: — Survey Questions. (DOCX 28 kb) [file 12890_2016_179_MOESM1_ESM.docx]

## Additional file 1: Survey Questions

1. **Do you hold a multi-disciplinary team meeting exclusively for the diagnosis of ILD in your hospital?**
2. **How frequently does your MDT meet?**
   1. More than once per week
   2. Every 1-2 weeks
   3. Every 3-4 weeks
   4. Less than every 4 weeks
3. **How many cases are discussed per meeting?**
   1. 1-5 cases
   2. 6-10 cases
   3. >10 cases
4. **How long does the meeting generally last?**
   1. 0-30 minutes
   2. 31-60 minuts
   3. 61-90 minutes
   4. >90minutes
5. 6**. From where does your MDT derive the cases it reviews?**
   - Cases managed by your centre’s clinical team
   - Cases exclusively managed and presented by clinical teams based at centres not associated with your centre
   - Cases managed at peripheral centres
   - Other (please specify)
6. **How many people generally attend your MDT meeting?**
7. **Which disciplines generally attend most meetings?**
   - Thoracic medicine
   - Radiology
   - Histopathology
   - Rheumatology
   - Immunology
   - Thoracic surgeon
   - Transplant physician
   - Palliative care physician
   - Nursing staff
   - Physiotherapy
   - Fellows/registrars
   - Junior trainees
   - Medical students
   - Others (please specify)
8. **Considering the attendees listed above, in your professional experience, which discipline/disciplines tend to have the most/least input at MDT meetings?**

|  | Always | Frequent | Seldom | Never | Does not attend |
| --- | --- | --- | --- | --- | --- |
| Thoracic medicine  Radiology  Histopathology  Rheumatology  Immunology  Thoracic surgeon  Transplant physician  Palliative care physician  Nursing staff  Physiotherapy  Fellows/registrars  Junior trainees  Medical students  Others (please specify) |  |  |  |  |  |

1. **Which one of the above disciplines listed above is responsible for leading the discussion in the MDT?**
2. **Which of the following best describes clinical data presentation at your MDT?**
   1. All clinical and investigation findings, whether or not relevant to the clinical case, are presented using a uniform template and detailed audiovisual presentation.
   2. Select clinical and investigation findings felt relevant by the clinical team are presented, in a detailed audiovisual presentation.
   3. Select clinical and investigation findings felt relevant by the clinical team are presented, in a detailed oral presentation.
   4. Select clinical and investigation findings felt relevant by the clinical team are presented, in a short oral presentation.
   5. Other (please specify).
3. **Which investigations are presented as a minimum routine requirement at all case presentations?**
   - Chest X ray
   - High resolution CT chest
   - Pulmonary function tests
   - Six minute walk test findings
   - Cardiopulmonary exercise test
   - Polysomnography
   - Surgical lung biopsy (if available)
   - Transbronchial biopsy (if available)
   - Broncho-alveolar lavage findings
   - Rheumatologic serology
   -

Biochemistry
   - Haematology
   -

Echocardiography
   - Right heart catheter study
   - Other (please specify)
4. **Who documents the final diagnosis and other decision making within the meeting?**
   1. The clinician (or their fellow/registrar) responsible for the case
   2. Other (please specify)
5. **Is a degree of diagnostic confidence assigned to the final diagnosis?**
   1. Yes
   2. No
6. **How many differential diagnoses are generally documented?**
   1. None
   2. 1-3
   3. >3
7. **When formulating a differential diagnosis, if there is disparity of opinion at the MDT, what process is used to determine the order of diagnostic likelihood?**

(free response)

1. **In the process leading up to the generation of a final diagnosis, is there one group that has a greater say in its formulation?**
   1. Yes. The clinician responsible for the patient's care.
   2. Yes. The chair of the MDT meeting
   3. No. We always make a consensus diagnosis.
   4. Other (please specify)
2. **After the final diagnosis is reached, is one group more accountable than others for that diagnosis?**
   1. Yes
   2. No

If you answered yes, which group are you referring to?

1. **What other information is specifically documented by the multi-disciplinary team?**
   1. Prediction of disease behaviour
   2. Management recommendations
   3. Treatment aims (with regards the best anticipated response to specific therapy)
   4. None of the above
   5. Other (please specify)
2. **List the three most common diagnostic or management dilemmas discussed at your MDT.**
   1. Dilemma 1
   2. Dilemma 2
   3. Dilemma 3
3. **Please provide any other comments you would like to make about MDT meetings?**
